# Complete neurologic and cognitive recovery after plasmapheresis in a patient with chronic inflammatory demyelinating polyneuropathy after allogeneic hematopoietic stem cell transplantation

**DOI:** 10.1007/s00508-016-0972-2

**Published:** 2016-02-26

**Authors:** Ursula Vogl, Gerda Leitner, Assunta Dal-Bianco, Marija Bojic, Margit Mitterbauer, Werner Rabitsch, Peter Kalhs, Axel Schulenburg

**Affiliations:** 1 Oncology, Medical Department I, St. Josef Hospital, Vienna, Austria; 2 Transfusion Medicine, Medical University of Vienna, Vienna, Austria; 3 Neurology, Medical University of Vienna, Vienna, Austria; 4 Bone Marrow Transplantation Unit, Department of Internal Medicine I, Medical University of Vienna, Währinger Gürtel 18–20, 1090 Vienna, Austria

**Keywords:** Autoimmune neuropathy, Allogeneic hematopoietic stem cell transplantation, Plasmapheresis

## Abstract

Neurologic complications after allogeneic hematopoietic stem cell transplantation (HSCT) are rare but poorly understood. We present a case report of a 57-year-old-male patient who was diagnosed in 2009 with acute myeloid leukemia (AML). He received two standard induction chemotherapies, as well as a following consolidation. Six months later, an allogeneic HSCT was performed. Shortly after HSCT the patient developed progressive polyneuropathy of the lower legs and hypoesthesia. Five months later a severe dementia followed. All images of the brain and spine showed no specific pathologies. High dose corticosteroids and immunoglobulins did not improve the neurologic symptoms. Due to severe worsening of the neuropsychiatric status and the clinical presentation, chronic inflammatory demyelinating polyneuropathy (CIDP) was suspected. Therefore, the patient received ten cycles of plasmapheresis. The patient showed a significant improvement of the neuropsychiatric symptoms and cognitive status. CONCLUSIONS: Immune mediated neuropathies after allogeneic HSCT, such as CIDP, have great variability in symptoms and presentation and are challenging to diagnose and treat. Plasmapheresis is a safe and efficient treatment for patients with unclear persisting autoimmune neuropathy after HSCT.

## Introduction

Neurologic complications ,such as chronic inflammatory demyelinating polyneuropathy (CIDP), myasthenia gravis or Guillain–Barré syndrome, after allogeneic HSCT are rarely seen but have great variability in symptoms and presentation and are challenging to diagnose and treat [1–3]. Plasmapheresis is established as effective and should be offered as short-term management of CIDP [4, 5] (Class I studies, level A).

## Case report

A 56-year-old-male patient was diagnosed in 2009 with an acute myeloid leukemia (AML; French–American–British FAB: M1, cytogentics: FLT3 neg, mDx Hema Vision Multiplex RT-PCR neg., Tryptase pos.) Initially he received standard induction chemotherapy with cytarabine, daunorubicin, etoposide, and a second induction chemotherapy with MIDAC (mitoxantrone, cytarabine) after blast cell persistance. The following consolidation was similar to the second induction chemotherapy (MIDAC). Six months later an HLA-identical unrelated donor was available. After conditioning chemotherapy, an allogeneic hematopoietic stem cell transplantation (HSCT) was performed. Conditioning chemotherapy consisted of Amsacrin 100 mg/m^2^, Fludarabine 30 mg/m^2^, and Cytarabine 2000 mg/m^2^ from days − 12 to − 9; after 3 days of rest, 4 Gy total-body irradiation (TBI) on day − 5; Thymoglobuline 2.5 mg/kg and Cyclophosphamide 60 mg/m^2^ on days − 4 and − 3. He received 6.3 × 106/kg body weight peripheral blood stem cells from an unrelated donor on June 17, 2009 [6]. Cylosporin A (CsA), along with mycophenolatmofetil, was used as an immunosuppressant. Leukocyte engraftment was observed on day + 14 (> 0.5 G/l) and platelets on day + 8 (> 20 G/l). During transplantation he experienced a bacterial infection with staphylococcus epidermis on day 1. On day 15 he developed grade III acute graft-versus-host disease (GvHD) of the skin, which was treated with high-dose corticosteroids 2 mg/kg and resolved on day 45.

Shortly after allogeneic HSCT and after appearance of acute GvHD (August 10, 2009, day + 58) the patient developed progressive tremor and disorientation while experiencing a cytomegalovirus (CMV) reactivation. Other medical reasons, such as thrombotic thrompocytopenic purpura (TTP) and drug toxicities were ruled out. CMV reactivation was successfully treated with ganciclovir for 14 days. Polymorphism chain reaction (PCR) tests showed negative results for CMV. However, neurological symptoms were still present, and more tests were run. Magetic Resonance Imaging (MRI) and liquor tests were negative, CsA was discontinued. Shortly after, the patient presented with progressive polyneuropathy of the lower legs and hypoesthesia on both feet.

Five months later the patient additionally developed a severe dementia with changes in personality and urinary retention. MRI and computed tomographies (CT) of the brain and spine showed no specific pathologies, the positron-emission tomography (PET) CT was also negative. The spinal fluid analysis showed slightly elevated cells with high protein levels and lymphocytic cells. All viral and bacterial diagnostics in the liquor were negative. The somatosensory evoked potentials (SSEP) were pathologic in concern of the lower right extremity. First-line therapy consisted of high dose corticosteroids and immunoglobulins. Due to severe worsening of the neuropsychiatric status and the results that were highly suspicious for chronic inflammatory polyneuropathy, the patient received ten cycles of plasmapheresis, which started on December 31, 2009.

During plasmapheresis, the patient showed a significant improvement of the neuropsychiatric symptoms. The cognitive status improved to almost normal.

During the follow-up period over the last 3 years, the patient is still in good health, the cognitive status is normal. There is no sign of neuromotoric deficiency. In June 2012 he again developed muscle cramps in the lower left limb. The electromyogram and muscle enzymes were negative. He received another cycle of immunoglobulins (2 g/kg/BW) for 5 days. The muscle cramps are still present but lower in frequency and the condition is improving.

He is even able to do physical workout again. Considering that the patient was running marathons this was an important improvement in quality of life. The last neurological examination did not show any motoric deficiencies. Moreover, he is still in complete hematologic remission of the AML.

## Discussion

Neurological complications following allogeneic HSCT are described in about 10–20 % in the literature, but immune-mediated neuropathies such as CIDP or Guillain–Barré syndrome are rare with an incidence of about 1–4 % [7, 8]. There are reports that the onset of these neuropathies correlates with the event of acute or chronic GvHD. Other theories suspect that the GvHD targets the nervous tissue itself [9]. Clinical features have great variability, and distinguishing acute-onset CIDP from Guillain–Barre syndrome is challenging. Similar to our patient, typical clinical presentations were paresthesia, progressive muscle weakness, sensory dysfunction, and impaired balance. The onset of these symptoms was shortly after development of acute GvHD of the skin on day 45 after allogeneic HSCT. Usually the disease responds quite well to corticosteroids or high dose immunoglobulins. In our patient the symptoms worsened and another symptom, namely, decreased cognitive potential (dementia) appeared. Decreased cognitive potential and aggressive behavior are typical signs of dementia but not CIDP. One could discuss that this was a direct symptom of GvHD of the nervous tissue or brain tissue.

The pathogenesis that causes autoimmunity to nervous tissue such as pathogenic autoantibodies or a single triggering antigen is not yet known. Various infections have been implicated as triggering events but not yet proven [10]. In our patient a CMV reactivation happened shortly before the beginning of neurologic symptoms.

Plasmapheresis, which removes pathogenic antibodies or circulating immune factors have shown to be effective in treating CIDP in controlled studies [4, 5]. In our patient not only the peripheral neurologic symptoms but also the neuropsychiatric symptoms presenting as dementia resolved. After several years of follow-up our patient had no relapse of CIDP, had normal cognitive function and is still in complete hematologic remission.

In conclusion, CIDP is a rare complication after HSCT and is very often associated with acute GvHD. Plasmapheresis should be considered as an option in patients who do not respond to corticosteroids or immunoglobulins. Plasmapheresis provides a safe and quick relief to majority of patients with CIDP.
